# Supplementary material for: Yiai Fuzheng decoction inhibits triple-negative breast cancer by remodeling the immune microenvironment
Source: Front Immunol. 2025 Sep 30;16:1615631. doi: 10.3389/fimmu.2025.1615631 (PMC12518410; doi:10.3389/fimmu.2025.1615631)
Supplement: Supplementary file 2 [file DataSheet1.zip › Supplementary File 2/Treatment/C_vs_D_vs_H/02_PCA/All_Groups/PCA_Score_2D_Label.pdf]

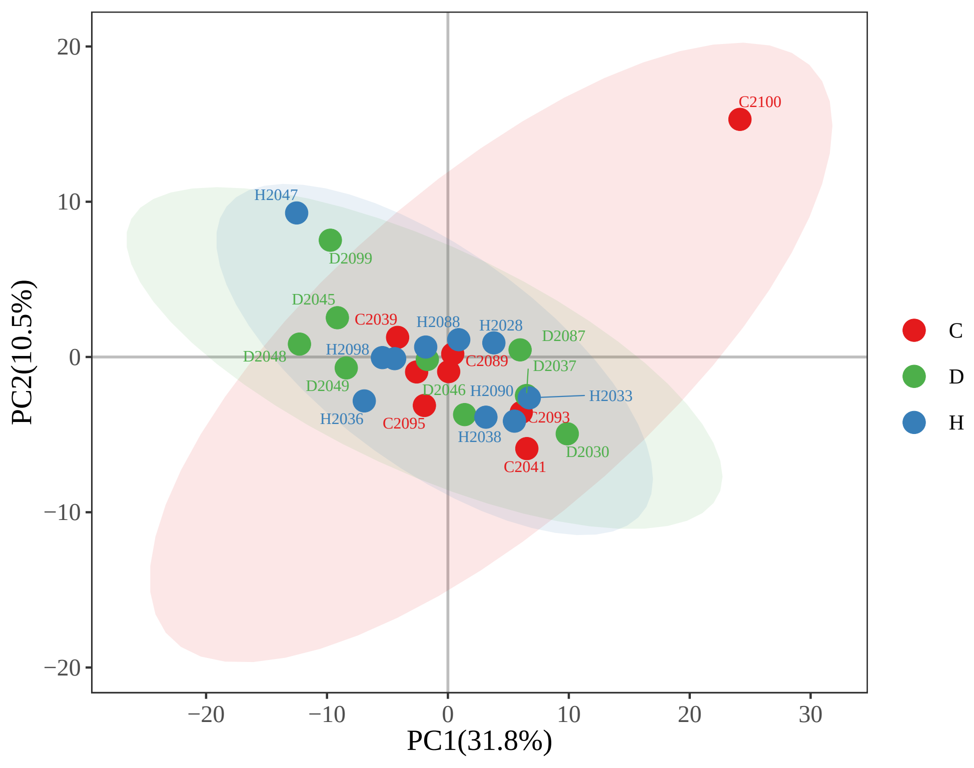
C2100
H2047
D2099
D2045
C2039
H2088
H2028
D2087
D2048
H2098
C2089
D2037
D2049
D2046
H2090
H2033
H2036
C2095
C2093
H2038
D2030
C2041
PC1(31.8%)
PC2(10.5%)
C
D
H
